# An overview of civic engagement tools for rural communities

**DOI:** 10.12688/openreseurope.18077.1

**Published:** 2024-09-06

**Authors:** Jorge Martinez-Gil, Mario Pichler, Noemi Lechat, Gianluca Lentini, Nina Cvar, Jure Trilar, Antonio Bucchiarone, Annapaola Marconi

**Affiliations:** 1 Software Competence Center Hagenberg GmbH, Softwarepark 32a Hagenberg,, 4232, Austria; 2 ADRETS, 69 rue Carnot Gap, 05000, France; 3 Poliedra-Politecnico di Milano, G. Colombo 40 Milano, 20133, Italy; 4 University of Ljubljana, Trzaska cesta 25 Ljubljana, 1000, Slovenia; 5 Fondazione Bruno Kessler Via Sommarive, Povo, Trento, 18,38123, Italy

**Keywords:** Smart Communities, Smart Villages, Civic Engagement

## Abstract

In this research, we explore the role of civic engagement platforms as tools designed to connect various groups in rural areas for collaborative advancement and to support sustainable growth in their communities. We examine these platforms’ essential features and influence on rural communities, conducting an overview to identify rural areas’ primary challenges and the functionalities needed to address them. Our findings reveal that the long-term capability of these civic engagement platforms can bring beneficial changes in rural territories by offering a unified way of communication, collaboration, and decision-making. The study concludes with suggestions for future research.

## Introduction

Recently, there has been an increasing interest in civic engagement solutions to facilitate active participation in communities ( Martinez-Gil *et al.*, 2022). Despite the abundance of platforms available for civic engagement, their application in rural areas remains unexplored ( Whitacre & Manlove, 2016). Our experience suggests that these tools can effectively support collaborative strategies, helping rural communities address challenges they are currently facing, like depopulation and diminished public services.

In the context of this work, we define *smart community* as a rural-based network whose members are connected by shared interests. At the heart of such a community is the concept of a *smart village*, which we have previously shown to be a rural settlement that leverages technology to improve residents' quality of life or economic opportunities ( Martinez-Gil *et al.*, 2019). This includes access to digital services and other aspects of importance in the rural world, such as agricultural automation, renewable energy, etc. This can usually be achieved through technologies such as IoT ( Liu *et al.*, 2019) and other technologies ( Hoch *et al.*, 2024). Therefore, this concept aims to facilitate technology to overcome critical challenges and facilitate rural territories' economic growth and sustainability ( Rozanova *et al.*, 2008).

However, the smart village concept is continuously evolving, and developments in this field are in constant flux. In this way, technology dedicated to civic engagement holds considerable promise. Civic engagement is about individuals participating in their communities, and technology can make this participation more accessible and practical ( Bringle *et al.*, 2011). One crucial yet often neglected aspect is developing civic engagement technology in rural communities, which frequently lack technological resources and face critical challenges. This is different, for example, in the context of cities where more research has been carried out ( Deng & Fei, 2023) ( Korten, 1996) ( Romano *et al.*, 2016) ( Smith, 2014), perhaps because of the abundance of data and the potential economic benefits of operating in such environments.

Therefore, while there has been a recent increase in civic engagement platforms, only some studies have explored their impact on rural communities. These platforms should strengthen ties between residents and their rural communities, giving them a voice and promoting dialogue on issues affecting their lives ( Min, 2007). They should offer the possibility of overcoming recurrent obstacles in rural areas and help residents decide about their community's future ( Mindel *et al.*, 2016). Therefore, civic engagement platforms could lead to collaborative strategies for addressing many rural challenges ( Thompson, 2021).

Our goal in this study is to provide an overview of the civic engagement platforms in rural settings. We aim to contribute to developing these platforms and outline their significance in rural communities. To this end, we have reviewed existing literature on civic engagement platforms and provided the following contributions:

- This research examines the critical features of civic engagement platforms, focusing on rural areas. It will assess how these platforms can support civic engagement. This study will provide insights into the practical challenges encountered while implementing these platforms and propose effective solutions, as detailed in Section 3.
- Additionally, this research will identify the challenges associated with these platforms and discuss their potential impact on rural communities. It will investigate how technology can help promote civic participation, which will be elaborated in Section 4.
- Furthermore, this study will address these platforms' potential risks and disadvantages, including concerns about the possibility of their misuse for harmful purposes. These issues will be thoroughly explored in Section 5.

Beyond the sections above, Section 2 will critically review current civic engagement tools and evaluate their effectiveness in rural settings. Section 6 concludes our overview by summarizing the most important findings and proposing a clear direction for further research.

## State-of-the-art

One area where technology has the potential to have a significant impact is civic engagement ( Quinn & Ramasubramanian, 2007). Civic engagement means individuals participating in their community, and technology can make this process easier and more accessible than ever before ( Bringle *et al.*, 2011). Despite years of study and exploration, one area that has been overlooked is the technology for civic engagement in rural areas.

The literature identifies several strategies to prevent recurring problems by making these areas more attractive and viable places to live and work ( Zavratnik *et al.*, 2018). These strategies include improving access to education, healthcare, and other essential services (e.g., sustainable agriculture, rural tourism, or promotion of local businesses). In addition, initiatives encouraging and supporting remote work could help sustain those rural communities. Several works have addressed this problem, building upon foundational approaches to the challenge ( de Zúñiga & Valenzuela, 2011) ( Hall, 1986) ( Rozanova *et al.*, 2008) ( Thompson, 2021). However, more studies still need to be conducted on the potential impact of using civic engagement platforms in a rural context.

In addition, it is also necessary to note that civic engagement platforms are online platforms designed to increase people's involvement in collaborative processes ( Cvar *et al.*, 2020). These platforms provide a space where people can express their opinions, share their experiences, and discuss different issues that they consider necessary ( Datyev *et al.*, 2020). Therefore, civic engagement platforms have the potential to become an essential tool for enabling people to participate in many short-term but also long-term strategies ( Desouza & Bhagwatwar, 2014).

An essential requirement of these platforms is that they should be designed to be inclusive so that all backgrounds are represented and can participate ( Chan, 2018). This can increase representation and guarantee that the opinions of minorities are also considered. Furthermore, these platforms can shed light on the decision-making processes and help provide a space for fruitful discussions.

Another essential aspect of civic engagement platforms is that they can promote a sense of community among people ( Alayed & Lutters, 2015). The idea is to help increase social cohesion ( Boulianne, 2023). This is important as it can promote a sense of belonging and responsibility for the community's well-being ( Smith, 2014).

Based on our literature analysis, it is possible to state that a civic engagement platform for smart rural communities should promote rural engagement, ensuring accessibility to those with limited internet access or digital literacy ( Harding *et al.*, 2015). This could be done through offline tools and digital training ( Boardman *et al.*, 2011). It should cultivate a community spirit, enabling rural residents to connect and discuss local matters. In this sense, people should be encouraged to contribute ( Hofmann & Pappas, 2021). It should also promote a data-driven approach and gather insights on rural needs and preferences. Although optional, its integration with local government could also fill the gap between rural stakeholders and their elected officials.

Other interactive features like surveys, polls, and forums can facilitate decision-making. The platform should also recognize linguistic diversity and support multiple languages, ensuring inclusivity ( Chen *et al.*, 2012). Lastly, it should also deliver relevant content on critical issues such as local economic, environmental, and social matters directly impacting rural life.

In addition to these core features, a civic engagement platform for smart rural communities should be user-friendly, secure, and accessible on various devices, including smartphones and tablets; usually, web environments are easily adaptable to a wide variety of means of access ( Thomas, 2010). These features aim to ensure that the opinions of the rural residents are considered. Furthermore, if we focus on more academic-oriented studies conducted in this context, we can highlight the following:

- Robertson comprehensively examines the complex interplay between social media platforms and civic engagement, exploring its historical origins, theoretical foundations, and the practical consequences of this relationship ( Robertson, 2018). Furthermore, a similar line of research has also been extensively studied and analyzed by ( Warren *et al.*, 2013).
- Hanna and Ashby explored the design fiction to explore a new concept for a community engagement platform that blends various technologies. This process led the authors to modify their original design, focusing on a voice user interface and influencing the prototype's design ( Hanna & Ashby, 2016).
- Martinez-Gil *et al.* describe research on a framework for checking the smartness maturity level of villages in different areas, such as Mobility, Governance, Economy, Environment, Living, and People. The framework is intended to serve as a decision-making tool for various users and has been implemented and evaluated in several municipalities in the European Alpine space. The authors hope to continue improving the framework based on stakeholder feedback ( Martinez-Gil *et al.*, 2019).
- Beranič *et al.* discuss the development of a Digital Platform as part of the SmartVillages Project to aid the efficient transformation of rural areas into smart villages. Four key functionalities were identified for the platform: Self-assessment, Best Practices, Matchmaking, and Collaboration. The work outlines the specifications, interaction, and position of each functionality in the platform architecture. The platform can be adapted for use in other domains, such as cities, with the essential functionalities adapted to fit the smartness dimensions of the domain ( Beranič *et al.*, 2019). This allows for customized smartness assessments and activities within any domain.
- Martinez-Gil *et al.* presented a framework for data analysis in smart villages to help rural authorities transition to a sustainable digitalization model to address their challenges. The framework includes tools such as data collection, recommendations for good practices, fake form detection, village clustering, similarity calculation between villages, and ranking creation. The authors believe this framework would help facilitate the transition to smart villages and plan to improve it over time with user feedback. Future work includes collaboration features to reflect reality more accurately through data analysis incorporating input from various sources ( Martinez-Gil *et al.*, 2020).
- Martinez-Gil *et al.* also presented the development of a platform to help local authorities transition into a new village model using digitalization and technology. The platform is designed for various audiences, including individuals, local authorities, industry, policymakers, and researchers. The platform aims to provide tools for sharing experiences and data analysis to support informed decision-making ( Martinez-Gil *et al.*, 2022).
- Hassan discussed the concept of gamification and its potential application in civic engagement platforms. Gamification involves using innovative elements to motivate users in non-gaming contexts. The author proves that gamification could benefit civic engagement platforms, as they often need higher engagement levels ( Hassan, Governments should play games: Towards a framework for the gamification of civic engagement platforms, 2017). Gamification is also the central topic of exploring what elements within mobile e-participation (m-participation) tools contribute to increased citizen involvement in urban governance ( Thiel, 2015a). The study specifically focuses on the potential of game elements like achievement badges, reward systems, and opportunities for social interaction. The goal is to understand how these elements foster continuous dialogue.
- Last, Stojanova *et al.* examined over one hundred policies, projects, programs, or actions related to rural development. From this analysis, the research presents key findings and makes future recommendations. These insights aim to guide future research in the field and inform policymakers at local, regional, national, and EU levels. The work outlines the significance of rural development and its policy implications ( Stojanova *et al.*, 2021).

In the following section, we intend to detail a strategy for creating and implementing successful civic engagement platforms in rural areas. These solutions can guarantee that every community is integrated into the digital era with adequate support. Additionally, they are incorporating gamification techniques into civic engagement platforms in a manner that can significantly boost user retention.

## Civic engagement features for rural communities

Rural territories face several problems, including population decline and lack of access to public services. Such problems can lead to a potential decrease in quality of life ( Stojanova *et al.*, 2022). The literature suggests that several strategies can be used to avoid these recurring problems. One of these strategies is implementing civic engagement platforms that promote a sense of community and ensure all opinions are, at least, heard. Below, we explain some of the features that would be desirable in this kind of platform.

### Short-term/operational features

A smart community (at least in a rural context) is an ecosystem where digital tools directly address the complex issues of rural life. Smart communities are intended to improve quality of life, boost economic potential, and promote sustainability by facilitating access to information and creating the conditions for active community engagement. Table 1 shows us current civic engagement platforms that usually include the following features:

**Table 1.** Features, Descriptions, and Benefits of Rural Engagement Platforms.

Feature	Description	Benefit
**Content customization**	To allow users to customize the content that is more relevant to their interests (e.g., agriculture, education, and local news information).	Information that meets the specific needs and interests of the community members.
**Community engagement**	To facilitate collaboration and promote information sharing and participation in virtual discussions.	Improved communication and collaboration within the community.
**Integration**	To integrate with other tools like online marketplaces and provide a straightforward suite of services.	Access to many services improving stakeholder convenience and efficiency.
**Mobile accessibility**	To be accessible on mobile devices, enabling access to resources from anywhere.	High accessibility and convenience for community members on the go.
**Multi-lingual support**	To support multiple languages to ensure accessibility for non-dominant language speakers.	Broader accessibility and inclusivity.
**Real-time data**	To incorporate real-time analytics and relevant information on crop prices, diseases, or weather forecasts.	Relevant information that helps community members to make decisions.
**User-friendly interface**	User-friendly interface accessible to people with little digital literacy.	Easier adoption and use by a wider range of community members.

Therefore, at least in the short term, the design of a civic engagement platform for a rural territory should be intensely focused on accessibility, customization, community engagement, and integrating real-time data and other digital tools.

### Long-term/strategic features

Civic engagement tools should also offer resources that help rural communities engage with each other and external stakeholders. The goal is to achieve long-term and sustainable outcomes and processes in a community-context environment. Many long-term or strategic features should be implemented in this regard. Table 2 shows us the most important ones, each with a description and its corresponding benefit:

**Table 2.** Table of Community Engagement Features, Descriptions, and Benefits.

Feature	Description	Benefit
**Community engagement principles**	Guidelines that show how engagement should be approached, with a focus on the coverage of the needs of the community members.	It ensures that engagement methods are ethical and aligned with community practices.
**Strategies for effective engagement**	Tailored communication, shared planning, and ongoing conversations to help build strong relationships within a diverse community.	It improves the effectiveness of engagement efforts, leading to more successful and sustainable results.
**Tools for measuring engagement effectiveness**	Tools including surveys and KPIs designed to evaluate the extent and impact of the activities.	It provides quantitative and qualitative indicators to improve engagement strategies over time.
**Identifying and analyzing stakeholders and their interests**	Identifying key community members (sometimes called local heroes) and understanding their influence.	It ensures an inclusive approach to engagement, considering diverse needs and viewpoints.
**Developing an evidence-based community program**	Creation of community programs based on empirical evidence and best practices tailored to the needs of the rural community.	It increases the likelihood of program success due to the support of initiatives in local areas.
**Implementing the program**	Actual execution of community programs, involving resource allocation, activity coordination, and stakeholder collaboration.	It translates plans into action, leading to tangible improvements and progress within the community.

Therefore, long-term/strategic features should cover principles like inclusivity and alignment to local norms, plans for effective engagement through customized communication, measuring engagement effectiveness using feedback mechanisms, and processes for identifying and analyzing the stakeholders' participation. Additionally, these platforms should address the execution of evidence-based community programs.

### Gamification aspects

In the context of civic engagement platforms, the possibility of including gamification elements might help address the issue of low engagement levels that often undermine the intended purpose of these platforms ( Escobar & Vásquez-Urriago, 2014) ( Hassan & Hamari, 2020) ( Jiang *et al.*, 2021) ( Lehner *et al.*, 2014). Therefore, the goal is to motivate community members with game elements such as point systems, badges, or rewards to boost engagement levels ( Thiel, 2015b). Table 3 summarizes the most relevant gamification aspects most frequently implemented in platforms of this kind.

**Table 3.** Gamification Techniques for Civic Engagement Platforms for Rural Communities.

Technique	Description	How to Achieve it
**Leaderboards**	Using leaderboards to show top members supports healthy competition. This is effective for promoting a sense of community and recognition.	Implement a system to track user participation and contributions. Display a leaderboard to highlight top performers.
**Challenges**	Creating specific challenges for members. Completing these can earn points or rewards, increasing members' sense of accomplishment.	Develop a set of challenges relevant to the community. Reward members with points or other incentives for completing these tasks.
**Storytelling**	Encouraging members to share experiences related to the community. This can earn rewards and foster a sense of connection and empathy.	Create a platform feature for members to share their stories. Offer rewards for sharing and engaging with the stories of others.

When designing gamification strategies for rural engagement platforms, it is essential to consider rural communities' particular characteristics. Platforms should be created using intuitive design features, keeping limited internet access and low literacy rates in mind ( Hodges, 2004). Additionally, gamification strategies should incorporate local cultural elements to engage the local population better. The purpose is to improve participation levels while creating a sense of community, competition, and accomplishment that encourages stakeholders to contribute ( Rehm *et al.*, 2018).

### Role of motivational techniques

Motivational techniques are pivotal in driving participation ( Bassanelli *et al.*, 2022). In the context of civic engagement platforms this is typically done across the three phases: co-design, co-development, and co-delivery. The principal aim is to leverage gamification to enhance engagement and foster behavioral change within rural communities.

***Co-design phase.*** Motivational techniques ensure active participation and idea generation in the co-design phase, where stakeholders collaboratively conceptualize and design sustainable innovation processes. Integrating gamification methods and tools incentivizes involvement and serves as a comprehensive data collection and analysis mechanism. Through gamification, stakeholders engage in activities that facilitate understanding various aspects crucial for sustainable innovation. This includes assessing the state of an area, identifying available resources, mapping the skills of citizens, determining desired services, and evaluating the overall needs and potential of a rural area.

***Co-development phase.*** During the co-development phase, where the designed solutions are refined and developed, motivational techniques are instrumental in maintaining momentum and enthusiasm among participants. They continue to drive engagement by providing tangible incentives for continued involvement. Through gamification, tasks related to prototyping and testing are transformed into interactive and enjoyable experiences. Leaderboards, challenges, and stories incentivize active participation, fostering a sense of collaboration among stakeholders. Feedback loops and regular communication channels also ensure that participants remain motivated and invested in the co-development process.

***Co-delivery phase.*** In the co-delivery phase, where the implemented solutions are deployed and evaluated, the significance of motivational techniques in sustaining community participation and facilitating behavioral change cannot be overstated. Gamification-based methods serve as catalysts for driving adoption and adherence, encouraging individuals to embrace innovative practices and solutions. Through a motivation toolkit, participants are equipped with the resources and incentives necessary to engage in call-to-action activities and actively effect meaningful behavioral changes. Feedback mechanisms, reward systems, and social incentives reinforce desired behaviors, empowering communities to take ownership of rural innovations and drive sustainable change.

## Roadmap

Based on our experience, we have created a roadmap to build the ideal platform for boosting civic engagement in rural areas. Our strategic plan includes platform development, community participation, and measuring impact for long-term success, as shown in Table 4.

**Table 4.** Phases in the Development of Civic Engagement Platforms for Rural Communities.

Phase	Description	Outcome
**Platform Design**	This phase should focus on accessibility and compatibility across different devices. Implementation of strict security and privacy measures to safeguard user information.	Accessible and secure platform focusing on user experience, especially for rural communities.
**Community Engagement**	Engagement and community participation are vital. Collaboration between platform developers and community leaders to address local issues and priorities. Strategies should consider rural challenges, like language barriers, and include diverse participation methods.	Strong community involvement and tailored solutions addressing specific needs and challenges of rural communities.
**Sustainability**	Focus on developing sustainable business models for long-term viability. Utilize public-private partnerships for support. Regular evaluations to measure impact and identify improvement opportunities	Long-term, scalable solutions with measurable impacts, ensuring the continuous relevance and improvement of the platform.

**Figure 1.**
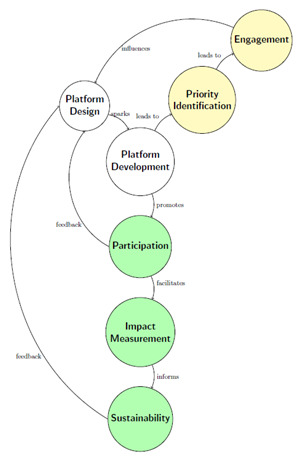
Life cycle concerning designing and deploying Civic Engagement Platforms for the rural world.

Figure 1 provides a flowchart illustrating the life cycle of building civic engagement tools in rural settings. This process is depicted through a series of interconnected nodes or processes, each representing a critical cycle stage. As can be seen, feedback loops must be frequent; otherwise, there is a risk of low participation of community members.

## Limitations

Civic engagement platforms hold promise in facilitating participatory activities, particularly within rural areas that might experience restricted accessibility to conventional forms of civic involvement. However, several challenges must be faced to ensure these platforms are effective ( DePaula *et al.*, 2018). Table 5 shows us some of the most critical obstacles to effectively operating such platforms.

### Risks and Limitations

**Table 5.** Risks/Limitations and Mitigations in Civic Engagement Platforms for Rural Communities.

Risk/Limitation	Description	Mitigation
**Digital Divide**	Not all rural areas might have reliable internet access to allow people to connect. This digital divide can further marginalize communities that are already disadvantaged ( Purdy, 2017) ( Whitacre & Manlove, 2016).	Implement alternative access ways such as offline capabilities and utilize local community centers for access.
**Limited participation**	Even if the infrastructure is available, platform participation can be low due to low digital literacy, lack of trust in the platform, or lack of interest. This can undermine the platform's effectiveness ( AlAwadhi, 2023).	Provide digital literacy training, guarantee transparency and security of the platforms, and engage communities in the design of these platforms.
**Contextual factors**	Many rural communities have distinctive cultural and social contexts that may need to be better fitted to existing civic engagement platforms. Designing platforms tailored to these communities' needs is essential ( Boulianne, 2023).	Collaborate with local leaders and community members to create and adapt platforms to local norms.
**Bias and representation**	Platform contents may be biased towards certain groups or may not adequately represent the diversity of their communities ( Uslaner & Brown, 2005). It is necessary to ensure that the platform represents the entire community.	Actively seek diverse perspectives in the development phase, implement features that promote diverse profiles, and regularly review content and user feedback for biases.
**Sustainability**	It might require resources to maintain, which may not be feasible in rural areas with limited resources considering the long-term sustainability and exploring alternative funding models ( Stojanova *et al.*, 2022).	Explore partnerships with NGOs, government agencies, and the private sector for support, and develop low-resource solutions.

## Discussion

Addressing all these problems might require a collaborative effort between platform developers, community organizations, and local authorities. Designing platforms that are accessible and tailored to the needs of rural communities promotes more effective engagement in these areas. Successful implementation of civic engagement tools involves a focus on platform design, community participation, and tracking impact for sustainability. It is also necessary to proceed with careful planning and collaboration between platform developers and community leaders so that effectiveness in the long term might be guaranteed.

## Conclusions and future work

This work has presented an overview of using digital technology to improve engagement and participation in rural communities. We have seen the potential benefits of implementing civic engagement platforms, such as increased collaboration, better access to information and resources, and facilitating informed decisions. We have also envisioned several challenges and constraints in developing these platforms, such as the need for digital literacy and access to technology.

Considering rural communities' unique context is essential when designing and implementing engagement platforms and for ongoing evaluation and adaptation. We can envision prospective directions for research and development in this area, including the possibility of transferring the lessons learned from one platform to another. Current technology has the potential to support more effective and inclusive engagement in rural communities, but it is essential to consider the stakeholder's needs in the design phase.

Future research should concentrate on developing more accessible and user-friendly civic engagement platforms. These platforms must effectively engage a broader spectrum of people, including those with limited digital skills and technology access. Potential strategies involve the creation of tailored training programs specific to supporting individuals who use these interfaces; furthermore, it is essential to design intuitive navigation systems for enhanced user experience.

Another area of future research could be to assess the long-term impact of smart community engagement platforms on rural communities. This could include conducting research studies to examine changes in community participation and collaboration over time due to the use of these platforms. Research could help identify critical factors contributing to these platforms' long-term sustainability.

To conclude, ongoing research to improve engagement and participation in rural communities has enormous potential to support more effective collaboration. However, to fully realize this potential, it is vital to consider rural communities' particular context and conduct permanent evaluation and adaptation to ensure their sustainability and effectiveness.

## Data Availability

No data are associated with this article.
