# Supplementary material for: An overview of civic engagement tools for rural communities
Source: Open Res Eur. 2025 Jan 31;4:195. Originally published 2024 Sep 6. [Version 2] doi: 10.12688/openreseurope.18077.2 (PMC11886549; doi:10.12688/openreseurope.18077.2)
Supplement: Peer review history

# Reviewer report: Reviewer response for version 2

**DOI:** 10.21956/openreseurope.21126.r50988

**Published:** 2025-03-05

**Authors:** Stavros Sindakis

**Affiliations:** 1 Hellenic Open University, Patras, Greece

The paper is a valuable contribution to the study of civic engagement in rural communities, highlighting the potential of digital platforms to enhance participation. However, it requires substantial improvements in theoretical grounding, methodological transparency, and evidence-based analysis. Strengthening these aspects will significantly improve the scientific rigor and impact of the study.

 1. Is the topic of the review discussed comprehensively in the context of the current literature?

 �� Analysis: The paper provides a broad review of civic engagement platforms, citing various sources and incorporating insights from previous studies. However, several gaps remain:

- Limited discussion on methodologies used in past research: The review lacks a structured approach in comparing existing civic engagement tools. There is no clear framework for evaluating these tools.
- Insufficient contextualization of key concepts: The definitions of civic engagement, smart villages, and smart communities are present but not deeply analyzed in relation to rural policy frameworks. The paper would benefit from stronger theoretical grounding in civic engagement research.
- Lack of reference to EU rural development policies: Since the study focuses on rural communities, incorporating references to EU smart village policies, rural sustainability strategies, or governance frameworks would strengthen its relevance.

�� Recommendation for Improvement:

- Introduce a comparative framework to analyze different civic engagement platforms.
- Expand the theoretical background by discussing smart community and smart village policies in-depth.
- Reference EU rural development policies to contextualize civic engagement in policy discussions.

2. Are all factual statements correct and adequately supported by citations?

 �� Analysis: While the paper includes a substantial number of references, several factual claims are not fully substantiated. For example:

- The section on gamification aspects states that gamification techniques "significantly boost user retention," but does not provide empirical evidence to support this claim.
- Some statements on the effectiveness of civic engagement platforms rely on author experience rather than peer-reviewed studies.
- The literature review does not systematically differentiate between theoretical contributions and empirical findings, making it unclear which aspects are evidence-based.

�� Recommendation for Improvement:

- Ensure that all claims about engagement effectiveness and platform benefits are supported by empirical research.
- Clearly differentiate between author insights and peer-reviewed findings.
- Provide case studies or specific examples where gamification has been successfully applied in civic engagement.

3. Is the review written in accessible language?

 �� Analysis: The article is mostly readable, but several issues hinder clarity:

- Repetitive sections: Some parts of the text, particularly in the Introduction and State-of-the-Art sections, restate information unnecessarily.
- Overuse of technical jargon: While the article is aimed at an academic audience, some complex terms (e.g., "civic engagement frameworks" and "data-driven participation") should be better explained.
- Unstructured presentation: The bullet-point format in the literature review (now changed in v2) still lacks a cohesive narrative flow.

�� Recommendation for Improvement:

- Remove redundant sections and ensure each paragraph presents unique insights.
- Define technical terms when first introduced.
- Improve flow between sections by using clear transitions and topic sentences.

4. Are the conclusions drawn appropriate in the context of the current research literature?

 �� Analysis: The conclusions summarize the findings well but lack critical reflection:

- The paper does not critically evaluate the limitations of civic engagement platforms beyond technical barriers.
- The potential risks of civic engagement platforms, such as data privacy concerns, digital exclusion, or misinformation, are minimally addressed.
- The recommendations for future research are too broad—it would be beneficial to suggest specific methodologies or case studies to explore.

�� Recommendation for Improvement:

- Include a critical discussion on the challenges of digital engagement beyond infrastructure and participation.
- Discuss potential unintended consequences, such as bias in engagement tools, privacy risks, or political misuse.
- Offer specific directions for future research, such as:
  - Investigating user behavior on civic platforms using mixed-method approaches.
  - Studying longitudinal impacts of engagement platforms on rural governance.
  - Examining the role of AI and automation in civic participation.

Is the review written in accessible language?

Partly

Are all factual statements correct and adequately supported by citations?

Partly

Are the conclusions drawn appropriate in the context of the current research literature?

Partly

Is the topic of the review discussed comprehensively in the context of the current literature?

Partly

Reviewer Expertise:

I specialize in innovation, entrepreneurship, knowledge management, and digital transformation, with a strong focus on academic publishing, ethical research practices, and technological applications in business and society. My expertise extends to civic engagement technologies, digital governance, and smart community development, making me well-positioned to provide a critical review of your paper.

I confirm that I have read this submission and believe that I have an appropriate level of expertise to confirm that it is of an acceptable scientific standard, however I have significant reservations, as outlined above.

# Reviewer report: Reviewer response for version 2

**DOI:** 10.21956/openreseurope.21126.r50621

**Published:** 2025-02-24

**Authors:** Magdalena Anna Zwolińska-Ligaj, Danuta Jolanta Guzal-Dec

**Affiliations:** 1 Faculty of Economic Sciences, John Paul II University in Biała Podlaska, Biala Podlaska, LUBELSKIE, Poland; 2 Faculty of Economic Sciences, John Paul II University in Biała Podlaska, Biała Podlaska, LUBELSKIE, Poland

The review addresses the current and significant problem of the role and possibilities of using digital technologies in the development process of rural communities. 

 The article is interesting but draws attention to several issues. It does not present a detailed research methodology and does not characterize the research material used. To ensure the academic validity of the text, these elements must be taken into account. The reader can guess, for example, that the authors performed some sort of structured review of selected platforms or a literature review on platforms, the results of which are included in tables. However, there is no knowledge of the criteria for selecting platforms for analysis, the methodological approach used, and finally - the number of platforms analyzed. It is therefore difficult to assess the scientific value of such a study. Care should be taken to ensure that all statements are supported by relevant and up-to-date references.

 Also, the theoretical review creates opportunities for enhancement. Key concepts such as civic engagement (appearing in the title of the work) were not explained with an indication of its relevance to the development of rural communities/smart rural communities, insufficient reference was made to the concepts of smart communities and smart villages (especially since these concepts were indicated as keywords). The theoretical basis/concept of policy formulation towards rural areas was not presented/indicated in the paper. It seems that by invoking the above concepts, it would be necessary to refer more extensively to the assumptions of the European Union's policy towards rural development - its conceptual/theoretical foundations (among others, sustainable development), including current definitions and key factors of rural development, including objectives, directions and, finally, policy tools with the indication of ICT technologies. Such a context seems necessary given the conclusions that can be drawn from Figure 1. It seems that Figure 1 needs more commentary. 

 Characteristics of civic engagement platforms need to be strengthened. It seems essential to provide a full characterization of civic engagement platforms, their features, diversity/types, possibilities for their use within the framework of rural development policy - how and by whom they can be used, what specific benefits can be obtained, the scale of their use and by which actors involved in the rural development process. The paper lacks references to research on existing platforms. 

 Additional remarks

 1. It is advisable to indicate the sources of the objects included.

 2. In some passages, the structure of the paper and the composition of the text are unreadable (formatting of sub-headings, section titles, placing objects directly after each other without commentary). 

 3. Consideration could be given to numbering the sections as there are references to section numbers in the introduction.

Is the review written in accessible language?

Yes

Are all factual statements correct and adequately supported by citations?

Partly

Are the conclusions drawn appropriate in the context of the current research literature?

No

Is the topic of the review discussed comprehensively in the context of the current literature?

No

Reviewer Expertise:

regional and local development, sustainable and smart development, innovation and entrepreneurship

We confirm that we have read this submission and believe that we have an appropriate level of expertise to confirm that it is of an acceptable scientific standard, however we have significant reservations, as outlined above.

# Reviewer report: Reviewer response for version 1

**DOI:** 10.21956/openreseurope.19537.r44131

**Published:** 2024-11-06

**Authors:** Lynn Schofield Clark

**Affiliations:** 1 University of Denver, Denver, Colorado, USA

I believe that there is some value to this article, but it is written in a repetitive way that makes it challenging to read. Because of a lack of information about existing systems, it is difficult to parse the specific contribution of this article. The piece needs significant editing to improve its clarity.

 For example, consider the section titled, State-of-the-art, which reads:

 “One area where technology has the potential to have a significant impact is civic engagement ( Quinn & Ramasubramanian, 2007). Civic engagement means individuals participating in their community, and technology can make this process easier and more accessible than ever before ( Bringle *et al.*, 2011). Despite years of study and exploration, one area that has been overlooked is the technology for civic engagement in rural areas.”

 This section comes immediately after the introduction, which already has provided a definition of civic engagement and already has noted the need for examination of civic engagement platform use in rural communities. The use of similar words (“one area”) and similar sentence structures makes it read as if written by AI.

 The initial literature review is written in bullet point format, which is unusual for an academic article and therefore fails to offer an overall summary of the literature in service of an argument. Several points are repeated (population decline in rural areas) and claims are made without support (“Table 3 summarizes the most relevant gamification aspects most frequently implemented in platforms of this kind,” although there is no evidence provided that suggests that these are the most frequently implemented aspects) .

 The discussion section is missing and the concluding section is inadequate.

 The title is inaccurate, as the article seems to be interested in presenting a rationale for a particular approach to the development of civic engagement tools for rural communities. It is not an "overview," as there are no tools that are reviewed, and in fact it is difficult to envision what, exactly, they have in mind because there are no examples discussed. It would be helpful, for instance, to discuss a particular tool that is used in urban communities and to then review how this would need to be modified for rural community use. This would help to clarify the particular contribution of this study. As it is, the features, descriptions, and benefits seem that they would apply equally well in urban, rural, or other settings in almost all cases. 

 My suggestion is that the authors read other academic articles in this area and model their writing after those. The authors also will want to be much more specific about what is lacking in existing tools, and what their model proposes to offer in the way of alternatives.

Is the review written in accessible language?

No

Are all factual statements correct and adequately supported by citations?

No

Are the conclusions drawn appropriate in the context of the current research literature?

No

Is the topic of the review discussed comprehensively in the context of the current literature?

No

Reviewer Expertise:

online civic engagement, platforms, digital studies

I confirm that I have read this submission and believe that I have an appropriate level of expertise to state that I do not consider it to be of an acceptable scientific standard, for reasons outlined above.

## References

[1] : Information technologies and civic engagement: Perspectives from librarianship and planning. *Government Information Quarterly* .2007;24(3) : 10.1016/j.giq.2006.08.005 595-610 10.1016/j.giq.2006.08.005

[2] : Designing Programs with a Purpose: To Promote Civic Engagement for Life. *Journal of Academic Ethics* .2011;9(2) : 10.1007/s10805-011-9135-2 149-164 10.1007/s10805-011-9135-2

# Response

**Published:** 2025-01-27

**Authors:** Jorge Martinez Gil

**Affiliations:** Software Competence Center Hagenberg, Austria

Dear Prof. Lynn Schofield Clark,

 Thank you very much for taking the time to read the manuscript and provide feedback. We understand that revisions are necessary, and we appreciate the chance to improve the work. We would also like to mention that this work is focused on the European Union, which may differ in certain respects from other regions of the world.

 1. We have cut back on reiterations and have now included details about existing systems in Section 2.

 2. We have noticed that some sections have repeated points made earlier. We have modified those passages, so each part has offered unique content, and the text now flows without unnecessary recurrence.

 3. We have transformed the bullet-point literature review into continuous prose. We have added references to support each claim. Please note, however, that we do not have references for some of the statements made in this paper as they are our own contribution and are based on over 8 years of experience building platforms for rural environments, please see: https://smart-villages.eu or https://smart-alps.eu. We acknowledge that this was not entirely clear in the first version, and we have worked to improve it.

 4. We have created a dedicated section that has discussed the findings. The final part has been revised to present what this manuscript has accomplished and what remains open for future work.

 5. We have introduced known civic engagement tools from other settings and have described how they could be adapted. We hope that this has made the intended contribution clearer and more concrete.

 Please note that we have not changed the title. This is problematic at this point as the preprint has been circulated, has had many downloads and has been cited in some technical reports. Changing the title could lead to a lot of confusion at this stage.

 6. We have examined other scholarly works in this area and have followed practices that have appeared commonly in related articles.

 We have explained where current tools have not met the needs of smaller communities and have shown how this work has addressed those gaps.

# Reviewer report: Reviewer response for version 1

**DOI:** 10.21956/openreseurope.19537.r44125

**Published:** 2024-09-24

**Authors:** Chanda Chansa Thelma

**Affiliations:** 1 Chreso University, Lusaka, Zambia

The study is significant as it explored the role of civic engagement platforms as tools designed to connect various groups in rural areas for collaborative advancement and to support sustainable growth in their communities. additionally, the study has presented an overview of using digital technology to improve engagement and participation in rural communities. it has also highlighted the potential benefits of implementing civic engagement platforms, such as increased collaboration, better access to information and resources, and facilitating informed decisions.

 However, the authors should work on tenses to suit the report format as it should be.

Is the review written in accessible language?

No

Are all factual statements correct and adequately supported by citations?

Yes

Are the conclusions drawn appropriate in the context of the current research literature?

Yes

Is the topic of the review discussed comprehensively in the context of the current literature?

Yes

Reviewer Expertise:

Civic Education, Civic Engagement, Citizenship.

I confirm that I have read this submission and believe that I have an appropriate level of expertise to confirm that it is of an acceptable scientific standard.
